# miR-141-3p promotes paclitaxel resistance by attenuating ferroptosis via the Keap1-Nrf2 signaling pathway in breast cancer

**DOI:** 10.7150/jca.96608

**Published:** 2024-09-03

**Authors:** Wan-Li Duan, Xue-Jie Wang, Ai Guo, Li-Hui Gu, Zhi-Mei Sheng, Hao Luo, Li-Xia Yang, Wen-Hao Wang, Bao-Gang Zhang

**Affiliations:** 1 Medical Research Center, Shaoxing People's Hospital, No.568, Zhongxing North Road, Shaoxing 312000, Zhejiang Province, China.; 2 Department of Pathology, Shaoxing People's Hospital, No.568, Zhongxing North Road, Shaoxing 312000, Zhejiang Province, China.; 3 Department of Pathology, Affiliated Hospital of Shandong Second Medical University, Weifang 261041, Shandong, China.; 4 Department of Medical Oncology, Affiliated Hospital of Shandong Second Medical University, Weifang 261041, China.; 5 Department of Diagnostic Pathology, School of Basic Medical Sciences, Shandong Second Medical University, Weifang 261041, China.

**Keywords:** miR-141-3p, Keap1-Nrf2 signaling pathway, breast cancer, ferroptosis, paclitaxel resistance

## Abstract

**Purpose:** Breast cancer poses a huge threat to the lives and health of women worldwide. However, drug resistance makes the treatment of breast cancer challenging. This study aims to investigate the effect of miR-141-3p on paclitaxel resistance and its underlying mechanisms in breast cancer.

**Methods:** Using bioinformatics analysis and qRT-PCR to explore the potential molecule miR-141-3p. Specific binding of miR-141-3p to Keap1 was determined by using a dual luciferase reporter assay. qRT-PCR and Western blot were utilized to observe the expression of miR-141-3p, Keap1, Nrf2, SLC7A11 and GPX4. GSH/GSSG content, MDA content and JC-1 assays were used to observe the ferroptosis levels of breast cancer cells. CCK-8 assay was used to observe the cell viability of breast cancer cells. Tumor subcutaneous transplantation experiment was used to understand the effect of miR-141-3p on paclitaxel resistance in breast cancer *in vivo*.

**Results:** In the present study, miR-141-3p was found to be highly expressed and associated with poor prognosis in breast cancer. miR-141-3p inhibited Keap1 expression, promoted Nrf2 expression, and facilitated paclitaxel resistance in breast cancer cells. Inhibition of miR-141-3p promoted Keap1 expression, inhibited Nrf2 and its downstream SLC7A11-GSH-GPX4 signaling pathway, as well as promoted ferroptosis in cancer cells, and inhibited paclitaxel and RSL3 resistance. ML385 blocks the effect of miR-141-3p on paclitaxel resistance and ferroptosis resistance in breast cancer cells. *In vivo*, miR-141-3p mimics promoted paclitaxel resistance, whereas miR-141-3p inhibitors inhibited paclitaxel resistance in breast cancer cells.

**Conclusion:** This work revealed that modulation of the Keap1-Nrf2 signaling pathway by miR-141-3p promoted paclitaxel resistance via regulating ferroptosis in breast cancer cells.

## Introduction

Breast cancer is the most common cancer in women and it poses a great threat to the health and life 1. The treatment of breast cancer includes surgical excision, radiation therapy, hormone therapy, chemotherapy and targeted biologic therapies 2. Unfortunately, drug resistance in breast cancer leaves patients with a poor prognosis and the possibility of relapse 3. Paclitaxel is one of the most commonly used first-line chemotherapeutic drugs for the treatment of breast cancer, however, resistance to paclitaxel can often be found in triple negative breast cancer, which creates a serious impediment to the treatment of breast cancer 4. Therefore, finding the specific mechanism of paclitaxel resistance is of great significance for the clinical treatment of breast cancer. Our study aimed to investigate the effect of miR-141-3p on paclitaxel resistance and its specific mechanism in breast cancer cells, further providing a theoretical basis for clinical treatment of breast cancer.

miRNAs, also known as microRNAs, play an integral role in multiple diseases and fields 5, 6. miRNAs often promote the degradation or inhibit the translation of mRNAs by targeting the 3'UTR of the target genes in cells 7. miRNAs have been shown to play an important role in tumor proliferation, invasion, metastasis and even drug resistance 5. Therefore, it is of positive clinical significance to explore the mechanisms by which miRNAs exert drug resistance in cancer cells and make interventions towards them.

Ferroptosis is a novel form of programmed cell death and distinguishes other forms of cell death. Ferroptosis is dependent on intracellular iron overload and accumulation of lipid hydroperoxides 8, 9. During tumor chemotherapy, it is often accompanied by ferroptosis of tumor cells, and focusing on the changes in ferroptosis level has a positive significance in understanding the drug resistance of tumor cells 10. A number of studies have demonstrated a positive effect on chemotherapy by promoting the onset of ferroptosis in tumor cells 11, 12. Cells resist ferroptosis mainly through four intracellular ferroptosis defense systems, including the glutathione-glutathione peroxidase 4 (GSH-GPX4) system in the cytoplasm or mitochondria, the ferroptosis suppressor protein 1-ubiquinol (FSP1-CoQH2) system in the cytosol, the dihydroorotate dehydrogenase-ubiquinol (DHODH-CoQH2) system in the mitochondria and the GTP Cyclohydrolase 1-tetrahydrobiopterin (GCH1-BH4) system that may be present in the cytoplasm 13. The GSH-GPX4 system is the most widely studied ferroptosis resistance system, and it plays a crucial role in cellular resistance to ferroptosis 13, 14. Solute Carrier Family 7 Member 11 (SLC7A11) is an upstream regulator of GSH-GPX4. As a membrane protein, its main function is to transport glutamate and cystine inside and outside the cell, and cystine can be used as a material to synthesize GSH and thus play an antioxidant role 15, 16. Taken together, SLC7A11-GSH-GPX4 is an important system for intracellular ferroptosis resistance.

Nuclear factor erythroid 2-related factor 2 (NRF2) is a nuclear transcription factor that promotes the transcription of various downstream antioxidant response elements in cells, including heme oxygenase-1 (HO-1), NAD (P)H: quinone oxidoreductase 1 (NQO1), GPX4, SLC7A11, etc., and plays a crucial antioxidant role in cells 17, 18. A number of studies have shown that Nrf2 has a key role in regulating ferroptosis in cells 19, 20. Kelch-like ECH-associated protein 1 (Keap1) binds to Nrf2 in the cytoplasm and promotes ubiquitination of Nrf2 for Nrf2 degradation during non-stressed conditions 21. When the cells are under stress, the binding of Keap1 with Nrf2 is disturbed and Nrf2 is released and translocated to the nucleus, thereby activating the transcription of downstream genes 22. Thus, regulation of Keap1 may influence the antioxidant effects exerted by Nrf2 and affect cellular resistance to ferroptosis.

miR-141-3p has been reported to have a role in promoting tumor progression in a variety of tumors, while miR-141-3p has been reported to have the ability to regulate Keap1-Nrf2 23-25. However, whether miR-141-3p regulates paclitaxel resistance in breast cancer and its specific mechanisms are not yet clear. This study aims to investigate whether miR-141-3p promotes paclitaxel resistance, as well as to explore the specific mechanism by which it promotes paclitaxel resistance in breast cancer, our finding may provide a new idea for clinical search of therapeutic target as well as chemotherapy for breast cancer.

## Materials and Methods

### Cell culture

MCF-10A, MCF-7 and MDA-MB-231 cell lines were purchased from Procell Life Science & Technology Co., Ltd. The above cell lines were cultured with DMEM medium containing 10% fetal bovine serum, 1% penicillin and streptomycin at 37°C in a 5% CO2 incubator. Cells were passaged at a 1:2 ratio when the cell confluence reached 85%-90%.

### Plasmid transfection and viral infection

Plasmid transfections: The miR-NC mimics, miR-141-3p mimics, miR-NC inhibitors and miR-141-3p inhibitors plasmids were purchased from GenePharma (Shanghai, China). Plasmid transfection was performed according to the instructions of Lip2000 (11668019, Thermo). 1×10^5^ cells were planted in six-well plates, the normal medium was changed to Opti-MEM medium when the cell confluence reached 70%-80%, and the cells were incubated in the incubator at 37°C for 30 minutes. The DNA-Lip complex was added into the cells (for MCF-7, the mass ratio of plasmid:Lip2000 was 1:1; for MDA-MB-231, the mass ratio of plasmid:Lip2000 was 1:0.8) and incubated at 37°C for 6h, subsequently, the culture medium was changed to the normal culture medium and incubated with incubator. RNA was extracted after 24h of transfection for qRT-PCR, and proteins were extracted after 48h of transfection for Western blot. Cells were treated with paclitaxel (for MCF-7, paclitaxel concentration was 20nM; for MDA-MB-231, paclitaxel concentration was 30nM) and ML385 (ML385 concentration was 2μM) 24-36h after plasmid transfection.

Viral infections: Viral vectors of miR-NC mimics, miR-141-3p mimics, miR-NC inhibitors, and miR-141-3p inhibitors were constructed at GenePharma (Shanghai, China). 2×10^5^ MDA-MB-231 cells were planted in 60mm medium dish, when the cell confluence reached 70%-80%, 2×10^7^TU/ml of viral vector was added to infect the cells. After 12h, the culture medium was replaced to normal medium, and the cells were cultured for 3 days and then added with 0.3 μg/ml puromycin for 4 days to obtain the stabilized transient cell line, which was used for the subsequent experiments of mouse subcutaneous tumor.

### Dual luciferase reporter assay

The binding site of miR-141-3p to the Keap1 3'UTR was predicted using the Targetscan website. Keap1 3'UTR wild-type (WT) and Keap1 3'UTR mutant (MUT) plasmid vectors were constructed at GenePharma (Shanghai, China). The above plasmids were co-transfected with the plasmid vector of miR-141-3p mimics into 293T cells, and the fluorescence intensity was detected by using a luciferase kit after 36-48 hours.

### qRT-PCR assay

For qRT-PCR of miRNA, the extracted RNA samples were reverse transcribed to cDNA using the M5 Stem-loop miRNA cDNA Synthesis Kit (MF878-01, Mei5Bio, Beijing, China) and PCR was performed with the Stem-loop miRNA Fluorescence Quantitative Detection Kit (MF879, Mei5Bio, Beijing, China), PCR reaction conditions were performed according to the instruction.

For qRT-PCR of mRNA, the extracted RNA samples were reverse transcribed using PrimeScript™ RT Master Mix (RR036A, Takara) reagent and detected by PCR using 2X M5 UltraSYBR Mixture (Low ROX) (MF015, Mei5Bio, Beijing, China) kit, PCR reaction conditions were performed according to the instruction.

PCR primers were synthesized by Sangon Biotech Co., Ltd. (Shanghai, China), and the primer sequences are shown in Table 1.

### Western blot assay

Total cellular proteins were extracted with pre-cooled total protein lysate (protease inhibitor and phosphatase inhibitor have been added in advance), Nuclear protein extraction was performed according to the instructions of the Nuclear and Cytoplasmic Protein Extraction Kit (P0027, Beyotime, Chine). Extracted proteins were used for protein concentration determination by using the BCA protein quantification kit (CW014, Kangwei, China), and heated at 100°C for 15 min in water bath after leveling. Proteins were separated by SDS-PAGE gel electrophoresis and transferred to PVDF membranes. PVDF membranes were incubated with 5% skim milk for 1h at room temperature followed by primary antibody incubation at 4°C overnight, subsequently, the membranes were washed three times with TBST and incubated with secondary antibody for 1h at room temperature. PVDF membranes were exposed after washed by TBST. Antibody informations are shown in Table 2.

### CCK-8 assay

Cell viability was assayed using Cell Counting Kit-8 (BS350B, Biosharp) following the procedures provided by the manufacturer. 3000-5000 cells were spread in 96-well plates and after transfection or drug treatment, CCK-8 solution was added to each well and incubated at 37°C for 1-4h. Subsequently, the 96-well plates were placed in microplate reader and the OD values were measured at 450 nm and cell viability was calculated.

### Measurement of GSH and GSSG content

Reduced glutathione (GSH) and oxidized glutathione (GSSG) are the two forms of glutathione. GSH is the main source of sulphhydryl groups in the vast majority of living cells and plays an important role in maintaining the redox state. GSH is a key antioxidant in cells, while GSSG is the oxidized form of GSH. We assayed the intracellular GSH content and the ratio of GSH/GSSG by using the GSH and GSSG Assay Kit (S0053, Beyotime Biotechnology). Experimental procedures were performed according to the steps provided by the manufacturer.

### Malondialdehyde (MDA) assay

MDA is the end product of lipid peroxidation, and the level of lipid oxidation can be effectively reflected by detecting the level of MDA. We assayed the MDA content of the cell samples using the Malondialdehyde (MDA) Content Assay Kit (BC0025, Solarbio). Experimental procedures were performed according to the protocols provided by the manufacturer.

### JC-1 assay

A decrease in mitochondrial membrane potential is one of the hallmarks of early apoptosis. We examined the mitochondrial membrane potential of MCF-7 and MDA-MB-231 cells by using the Mitochondrial membrane potential assay kit with JC-1 (C2006, Beyotime Biotechnology). Experimental protocols are provided by the manufacturer.

### *In vivo* tumorigenesis assay

A total of 21 4-week-old nude mice were purchased from Jinan Pengyue Laboratory Animal Breeding Co. Nude mice were housed in SPF-rated individually ventilated cages. The ambient temperature was 20-25°C, humidity was 50-60%, with sufficient water and food. Twenty-one nude mice were randomly divided into three groups: the MDA-MB-231+miR-NC mimics+miR-NC inhibitors+PTX group, the MDA-MB-231+miR-141-3p mimics+miR-NC inhibitors+PTX group, and the MDA-MB-231+miR-NC mimics+miR-141-3p inhibitors+PTX group. Each group was injected subcutaneously with 5×10^6^ stabilized MDA-MB-231 cells. Paclitaxel (20 mg/kg) was injected intraperitoneally every 2 days starting on day 10 and continued for 7 injections. Tumors were removed and quantified on day 30. All animal experiments were performed in accordance with National Institutes of Health guidelines and were approved by the Ethics Committee of Shandong Second Medical University (approval number: 2023YX069).

### Statistical analysis

Statistical graphs were plotted and statistical differences calculated using GraphPad Prism 9.0 software (GraphPad, San Diego, CA). All data are expressed as mean ± standard deviation. Student's t-test was used in order to compare the statistical significance of the two independent groups.

## Results

### miR-141-3p is highly expressed in breast cancer cells and specifically targets the 3'UTR of Keap1

**Figure 1:**
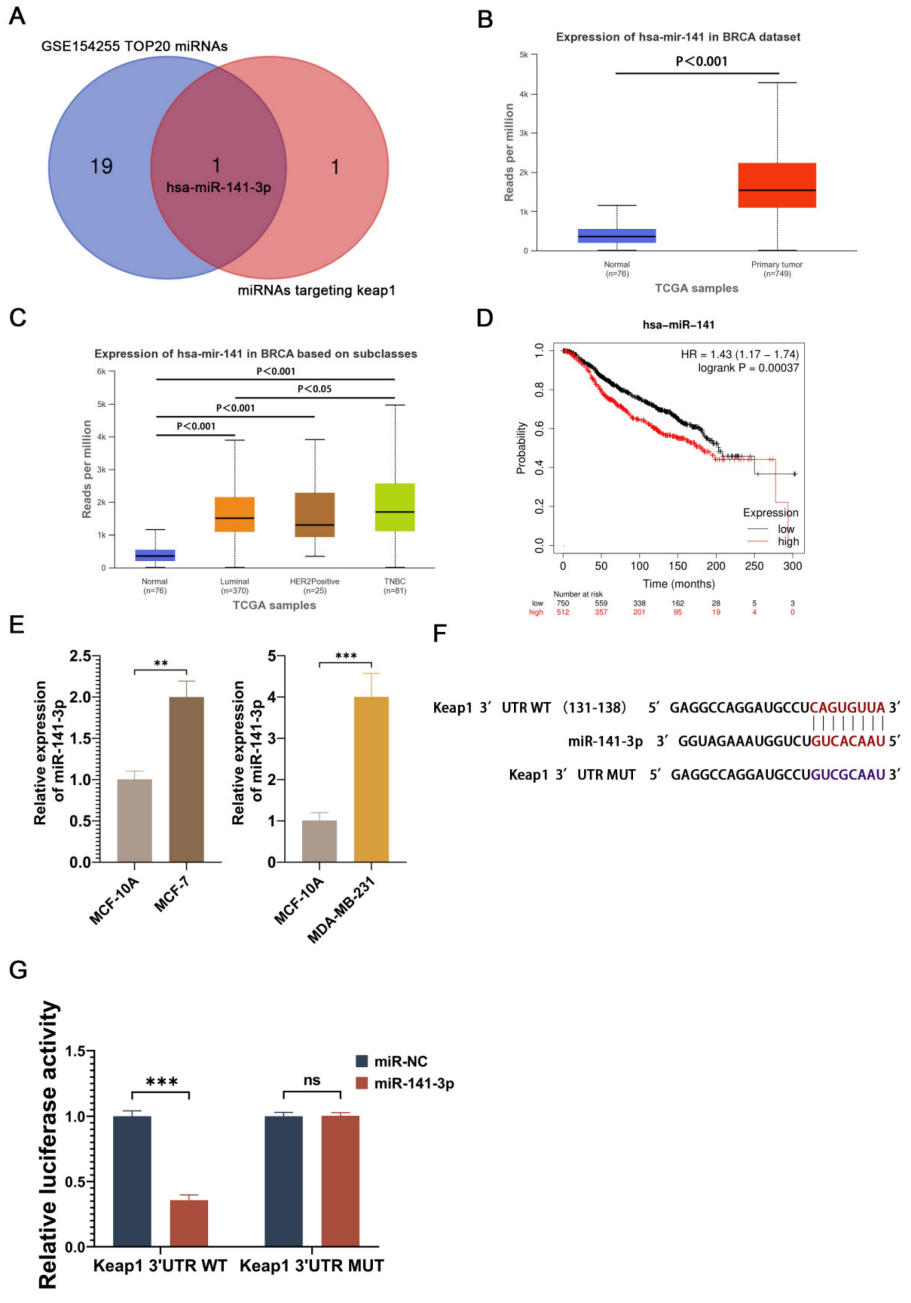
miR-141-3p was highly expressed in breast cancer and specifically targeted the 3'UTR of Keap1. (A) Twenty miRNAs significantly overexpressed in breast cancer in GSE154255 intersect with miRNAs that target Keap1. (B) TCGA database shows miR-141 is highly expressed in breast cancer. (C) Expression levels of miR-141 in different types of breast cancer in the TCGA database. (D) The relationship between miR-141 and survival of breast cancer patients. (E) Relative expression of miR-141-3p in MCF-7, MDA-MB-231 and MCF-10A cells as shown by qRT-PCR. (F) Prediction of miR-141-3p binding to Keap1 by using Targetscan. (G) Binding of miR-141-3p to Keap1 verified by dual luciferase reporter assay. **P < 0.01, ***P < 0.001, ns: no significance.

In order to find potential miRNAs that promote paclitaxel resistance in breast cancer cells, we performed the differential gene expression analysis of GSE154255 in the GEO database, which contains 10 cases of tumor tissues (3 triple-negative breast cancer tissues, 3 HER2-positive breast cancer tissues, 2 luminal A breast cancer tissues, and 2 luminal B breast cancer tissues) and 10 cases of the corresponding adjacent normal tissue. 48 miRNAs that were highly expressed in breast cancer were obtained using P<0.05 and logFC≧1 as the screening conditions. Meanwhile, Targetscan was utilized to search for miRNAs capable of targeting Keap1, and two potential miRNAs (miR-200a-3p and miR-141-3p) were obtained. The two miRNAs obtained from Targetscan were intersected with the 20 miRNAs that were most significantly different from the 48 highly expressed miRNAs screened in GSE154255 to obtain a potential miRNA (miR-141-3p) (Figure 1A). Analysis of miR-141 expression in breast cancer at the UALCAN website revealed that miR-141 was highly expressed in breast cancer (Figure 1B). miR-141 was highly expressed in different types of breast cancer and had higher expression in triple-negative breast cancer (Figure 1C). The survival prognosis of miR-141 in breast cancer was analyzed using the Kaplan-Meier Plotter website, and the results showed that high expression of miR-141 was associated with poor prognosis in breast cancer (Figure 1D). Although miR-141 does not completely represent miR-141-3p, miR-141-3p is sheared from the miR-141 precursor, and the expression and function of miR-141 may somewhat reflect the expression and function of miR-141-3p. In order to understand the expression of miR-141-3p in breast cancer, we compared the miR-141-3p expression in breast cancer cell lines (MCF-7 and MDA-MB-231) with the normal breast cell line (MCF-10A) by using QRT-PCR. Our results showed that the expression of miR-141-3p in MCF-7 and MDA-MB-231 was higher than MCF-10A (Figure 1D). Targetscan website showed that miR-141-3p binds at sites 131-138 of the Keap1 3'UTR (Figure 1E). Given that, we utilized a dual luciferase reporter assay to validate the binding site. The results showed that miR-141-3p could bind to the wild-type 3'UTR of Keap1, but not to the mutant 3'UTR of Keap1. The above data demonstrated that miR-141-3p is highly expressed in breast cancer cells and specifically binds to the 3'UTR of Keap1.

### miR-141-3p inhibits Keap1 expression and promote paclitaxel resistance in breast cancer cells

**Figure 2:**
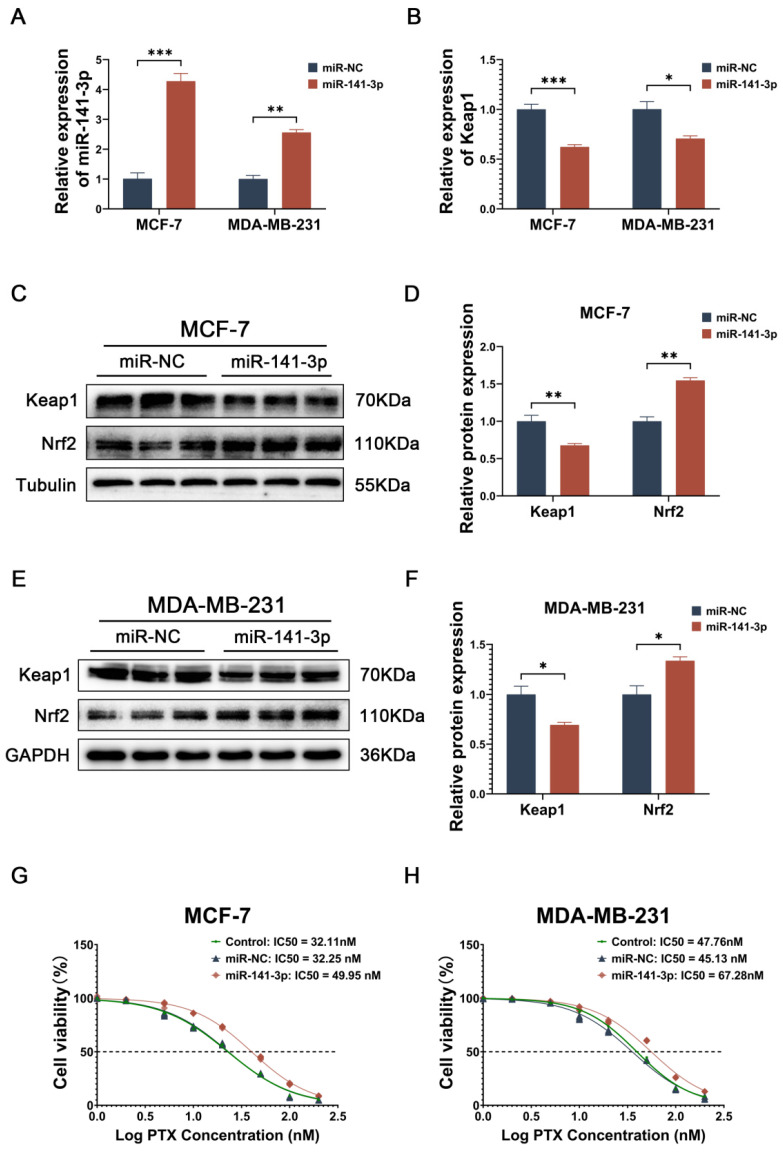
miR-141-3p mimics inhibit Keap1 expression and promote paclitaxel resistance in breast cancer cells. (A) Relative expression of miR-141-3p in breast cancer cells following transfection with miR-141-3p mimics plasmid as revealed by qRT-PCR. (B) Relative expression of Keap1 observed by qRT-PCR. (C-D) Western blot assay was used to detect the expression of Keap1 and Nrf2 in MCF-7 cells. (E-F) Western blot assay was used to detect the expression of Keap1 and Nrf2 in MDA-MB-231 cells. (G-H) IC50 of paclitaxel in different groups of MCF-7 and MDA-MB-231 cells. *P < 0.05, **P < 0.01, ***P < 0.001.

Subsequently, we observed the effect of miR-141-3p on paclitaxel sensitivity in breast cancer cells by overexpressing miR-141-3p. miR-141-3p was observed to be overexpressed in breast cancer cells transfected with miR-141-3p mimics (Figure 2A), and the mRNA level of its target gene, Keap1, was reduced (Figure 2B). Since Keap1 is a key protein in the regulation of Nrf2, and Nrf2 has an important antioxidant role in cells, we focused on the expressions of Keap1 and Nrf2 after overexpression of miR-141-3p in breast cancer cells. Western blot showed that after overexpression of miR-141-3p in MCF-7 cells, the expression of keap1 was down-regulated, while the expression of Nrf2 was up-regulated (Figure 2C-D), and the same result was shown in MDA-MB-231 (Figure 2E-F). We investigated the effect of miR-141-3p on paclitaxel resistance in breast cancer by comparing the half maximal inhibitory concentration (IC50) of paclitaxel on breast cancer cells, our results showed that the overexpression of miR-141-3p promoted paclitaxel resistance in breast cancer cells (Figure 2G-H). The above data suggested that mir-141-3p may promote paclitaxel resistance via the Keap1-Nrf2 signaling pathway in breast cancer cells.

### Inhibition of miR-141-3p promotes paclitaxel sensitivity in breast cancer cells

**Figure 3:**
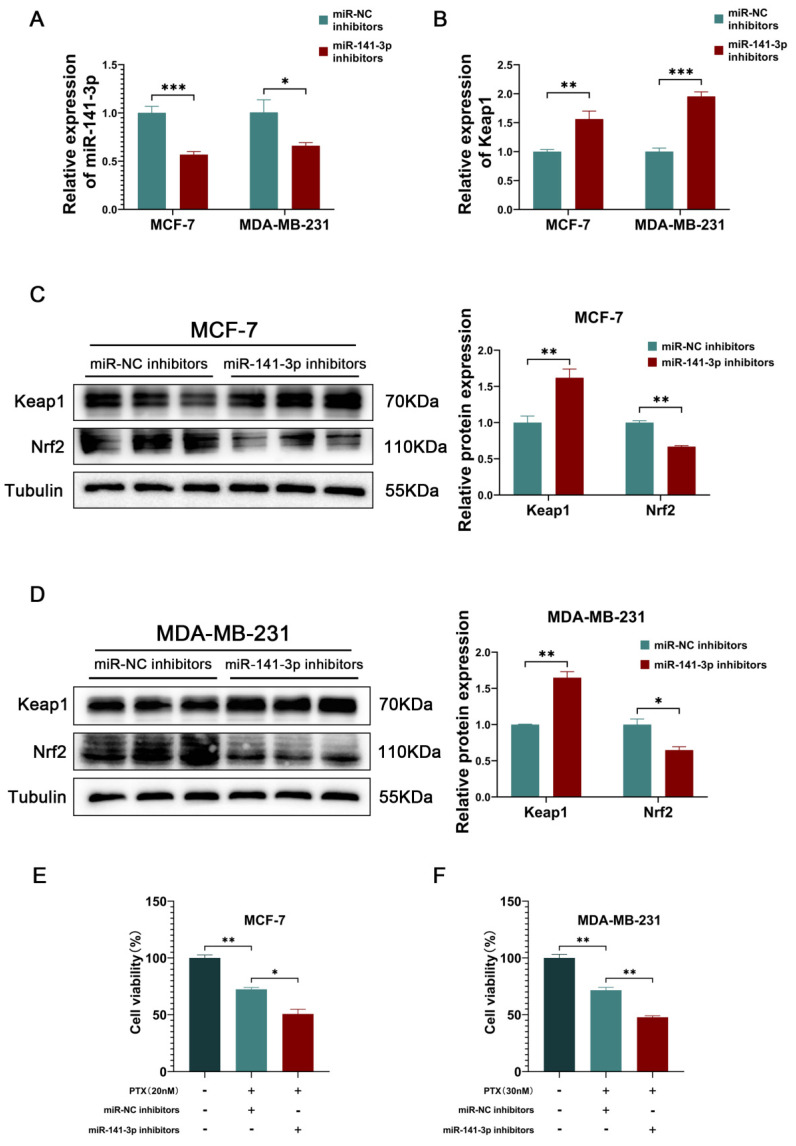
miR-141-3p inhibitors promoted paclitaxel sensitivity in breast cancer cells. (A) qRT-PCR experiments of miR-141-3p. (B) qRT-PCR experiments of Keap1. (C-D) The expression of Keap1 and Nrf2 in MCF-7 and MDA-MB-231 cells were observed by Weatern blot assay. (E-F) Cell viability of MCF-7 and MDA-MB-231 cells subject to paclitaxel treatment. *P < 0.05, **P < 0.01, ***P < 0.001.

Knowing that miR-141-3p promotes paclitaxel resistance in breast cancer cells, we hoped to inhibit paclitaxel resistance in breast cancer cells by targeting and inhibiting miR-141-3p. Therefore, the miR-141-3p inhibitors were utilized to target and reduce the expression of mir-141-3p (Figure 3A). The inhibition of miR-141-3p effectively increased the miRNA expression of Keap1 in cancer cells (Figure 3B). Western blot showed that miR-141-3p inhibitors increased the expression of Keap1 and decreased the expression of Nrf2 in MCF-7 cells, and the same results were shown in MDA-MB-231 (Figure 3C-D). CCK-8 assay was utilized to observe the effect of miR-141-3p inhibitors on paclitaxel resistance in breast cancer cells, and our results showed that miR-141-3p inhibitors suppressed paclitaxel resistance in MCF-7 and MDA-MB-231 (Figure 3E-F). These data demonstrated that inhibition of miR-141-3p promoted paclitaxel sensitivity in breast cancer cells.

### miR-141-3p inhibitors suppress ferroptosis resistance in breast cancer cells

**Figure 4:**
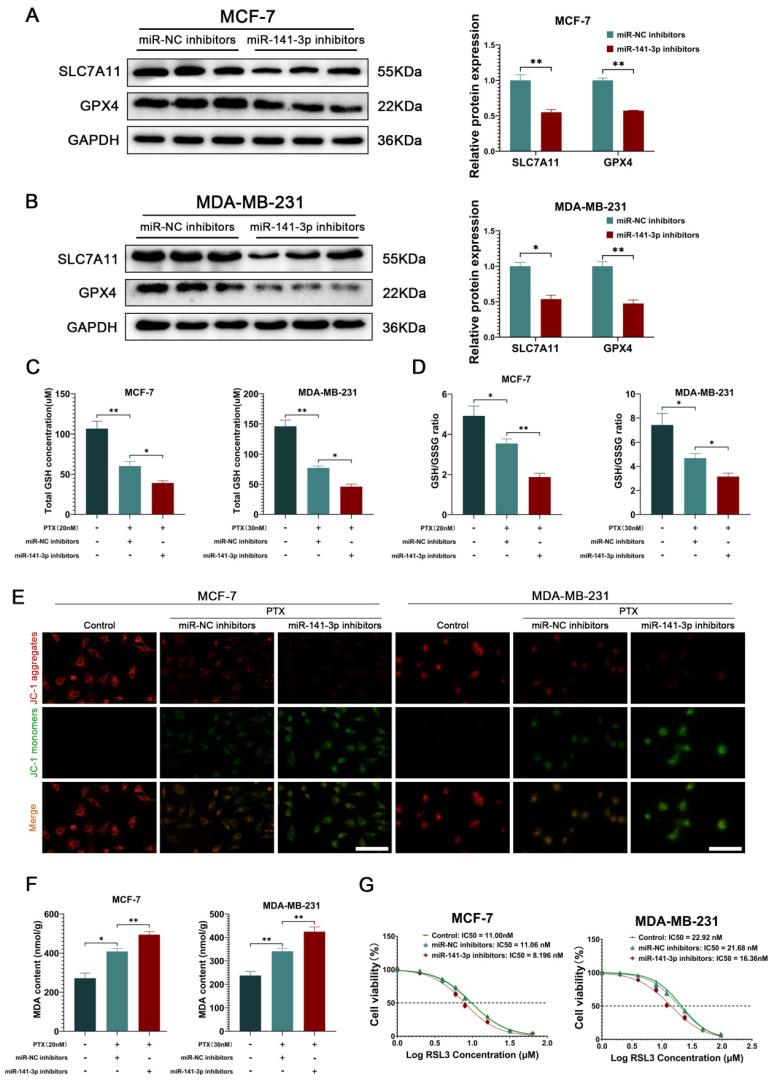
miR-141-3p inhibitors inhibited the ability of ferroptosis resistance in breast cancer cells. (A-B) The expression of SLC7A11 and GPX4 in MCF-7 and MDA-MB-231 cells were observed by Weatern blot assay. (C-D) Measurements of GSH and GSSG content in MCF-7 and MDA-MB-231 cells. (E) JC-1 experiment for visualizing the mitochondrial membrane potential. Scale bar = 50 μm. (F) Measurement of MDA content. (G) IC50 of RSL3 in MCF-7 and MDA-MB-231 cells. *P < 0.05, **P < 0.01.

Subsequently, we intended to explore the specific mechanisms by which miR-141-3p regulates paclitaxel resistance in breast cancer cells. Nrf2 has an important antioxidant role in cells as a nuclear transcription factor that promotes the transcription of downstream antioxidant components, including HO-1, GPX4, SLC7A11, and NQO1 20, 26, 27. Since the SLC7A11-GSH-GPX4 system is a classical intracellular ferroptosis-resistant system and ferroptosis resistance in cancer cells has been shown to be involved in chemoresistance in cancer cells 28, 29, we focused on the effects of miR-141-3p inhibitors on the SLC7A11-GSH-GPX4 system in cancer cells. Western blot showed that miR-141-3p inhibitors significantly decreased the expression of SLC7A11 and GPX4 in MCF-7 and MDA-MB-231 (Figure 4A-B). Meanwhile, we observed that miR-141-3p inhibitors further decreased the paclitaxel-induced GSH content and GSH/GSSG ratio reduction (Figure 4C-D). The ferroptosis is often accompanied by mitochondrial dysfunction, and we observed the mitochondrial membrane potential of the cells by JC-1 assay in response to the level of intracellular ferroptosis. Our results showed that miR-141-3p inhibitors significantly further reduced paclitaxel-induced reduction of the mitochondrial membrane potential in cancer cells (Figure 4E). Malondialdehyde (MDA) is the final product of intracellular lipid peroxidation, and MDA can effectively respond to the level of intracellular ferroptosis. Our results showed that miR-141-3p inhibitors significantly increased the level of MDA in paclitaxel-treated cancer cells (Figure 4F). Importantly, we observed that miR-141-3p inhibitors decreased the tolerance of RSL3, a ferroptosis inducer, in cancer cells (Figure 4G). The above results suggested that miR-141-3p inhibitors may inhibit paclitaxel resistance by promoting ferroptosis in breast cancer cells.

### ML385 blocks the facilitation of paclitaxel resistance posed by miR-141-3p in breast cancer cells

**Figure 5:**
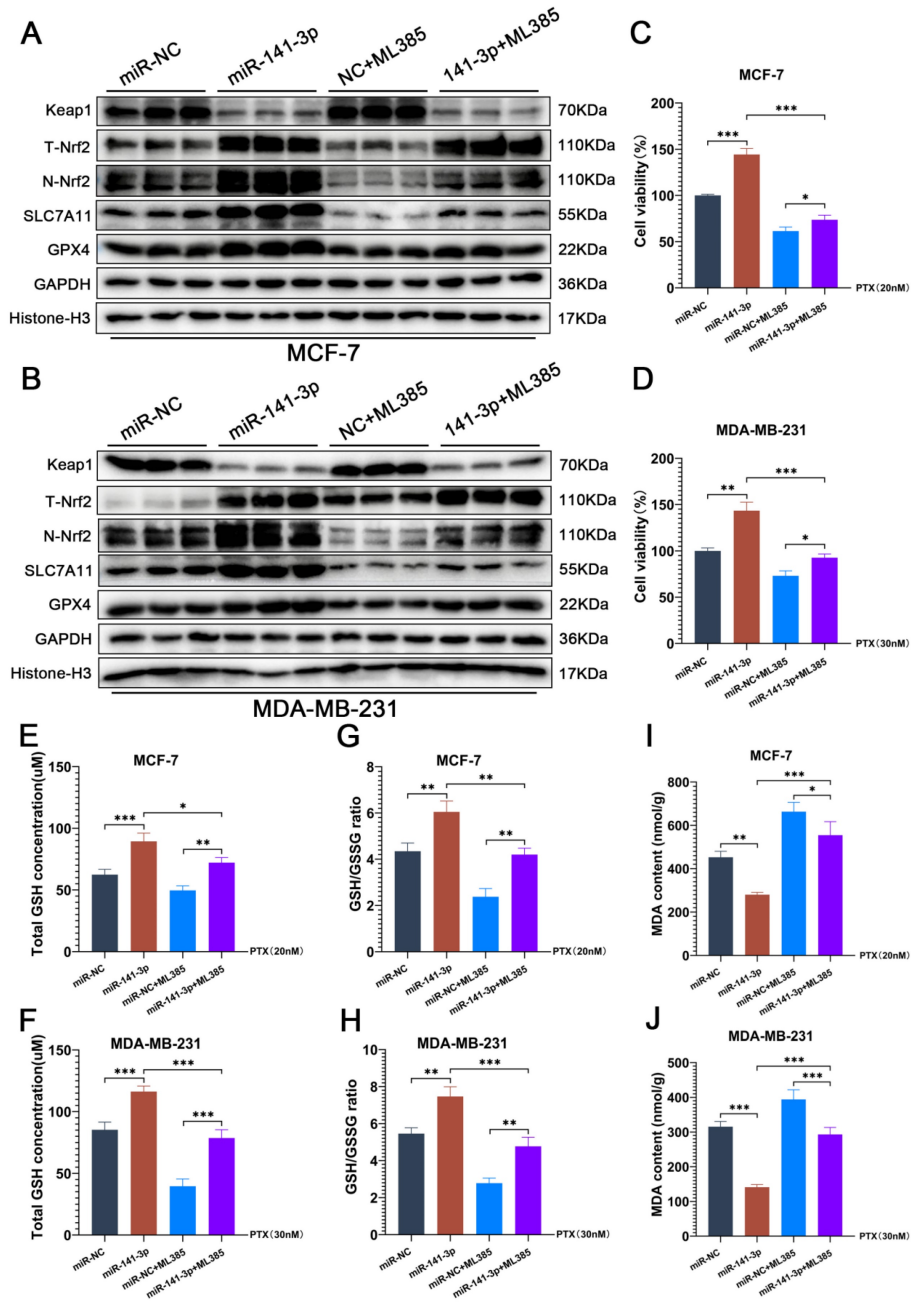
ML385 blocks the facilitation of paclitaxel resistance posed by miR-141-3p in breast cancer cells. (A-B) The expressions of Keap1, total Nrf2 (T-Nrf2), nuclear Nrf2 (N-Nrf2), SLC7A11 and GPX4 in MCF-7 and MDA-MB-231 cells were observed by Weatern blot assay. (C-D) CCK-8 assay was performed to observe the viability of MCF-7 and MDA-MB-231 cells that received paclitaxel treatment. (E-H) Measurements of GSH and GSSG content in MCF-7 and MDA-MB-231 cells. (I-J) Measurement of MDA content. *P < 0.05, **P < 0.01, ***P < 0.001.

To further determine that the promotion of paclitaxel resistance was mediated through the regulation of Nrf2 by miR-141-3p in breast cancer cells. We utilized ML385 to inhibit the transcriptional activity of Nrf2 and observed the changes of paclitaxel resistance and the ferroptosis levels in breast cancer cells. ML385 can interact with Nrf2 and significantly inhibit the binding activity of Nrf2 to DNA, which in turn inhibits the transcriptional ability of Nrf2. The results of our experiments showed that ML385 did not affect the expressions of Keap1 and total Nrf2 (T-Nrf2) in breast cancer cells, but significantly reduced the expression of Nrf2 in the nucleus (N-Nrf2) (Figure 5A-B). Meanwhile, ML385 significantly inhibited the enhancement of miR-141-3p on SLC7A11 and GPX4 expression (Figure 5A-B). CCK-8 experiments showed that ML385 reduced cell viability under paclitaxel treatment and blocked miR-141-3p-mediated paclitaxel resistance in breast cancer cells (Figure 5C-D). GSH and GSSG content assay showed that ML385 significantly suppressed the promotional effect of miR-141-3p on the GSH content and GSH/GSSG ratio in breast cancer cells under paclitaxel treatment (Figure 5E-H). MDA content measurements also showed that ML385 significantly aggravated the MDA content in breast cancer cells under paclitaxel treatment (Figure 5I-J). These results indicated that the inhibition of Nrf2 transcriptional activity by ML385 significantly blocked the promotion of miR-141-3p on ferroptosis resistance and paclitaxel resistance in breast cancer cells.

### miR-141-3p promotes the paclitaxel resistance of breast cancer cells *in vivo*

**Figure 6:**
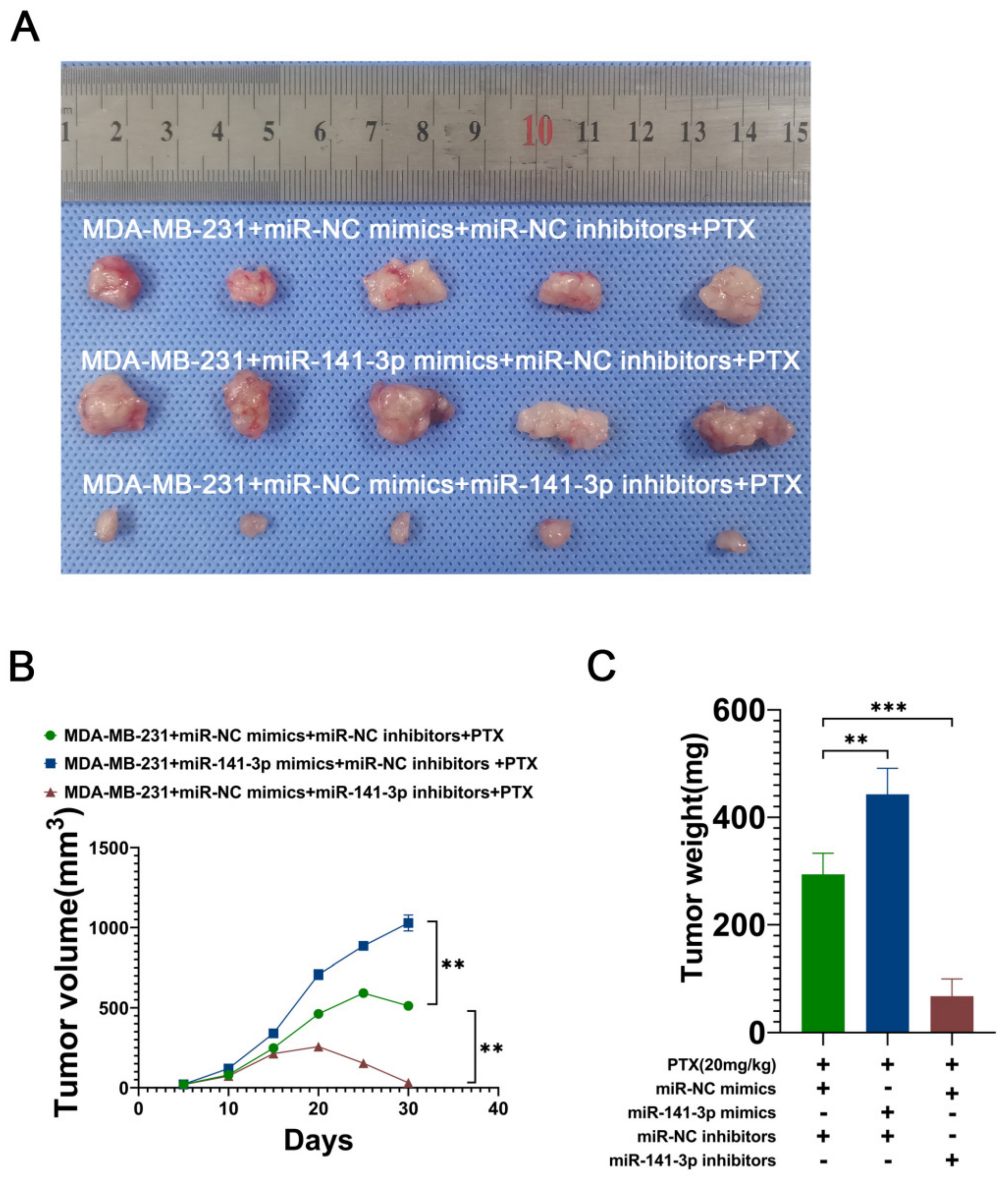
miR-141-3p promotes paclitaxel resistance in breast cancer cells in mice. (A) Representative subcutaneous tumor tissues (n = 7). (B) Quantification of tumor volume. (C) Quantification of tumor weight. **P < 0.01, ***P < 0.001.

To further demonstrate that miR-141-3p can promote the paclitaxel resistance of breast cancer cells *in vivo*, we utilized lentiviral constructs of stably transduced miR-141-3p overexpressing MDA-MB-231 cell lines (miR-141-3p mimics) and miR-141-3p underexpressing MDA-MB-231 cell lines (miR-141-3p inhibitors). The breast cancer model was established by subcutaneous implantation of MDA-MB-231 in nude mice, and 10 days later continuous intraperitoneal injection of paclitaxel (20 mg/kg) was used to observe the drug resistance of breast cancer cells in mice. Mice were divided into three groups: the MDA-MB-231+miR-NC mimics+miR-NC inhibitors+PTX group, the MDA-MB-231+miR-141-3p mimics+miR-NC inhibitors+PTX group and the MDA-MB-231+miR-NC mimics+miR-141-3p inhibitors+PTX group. Our results showed that MDA-MB-231 cells overexpressing miR-141-3p had higher paclitaxel resistance in mice compared to the control group, with a larger tumor volume and heavier tumor weight, and inhibition of miR-141-3p expression resulted in higher paclitaxel sensitivity, smaller tumor volume, and lighter tumor weight (Figure 6A-C). These data suggested that miR-141-3p promotes the paclitaxel resistance of breast cancer cells *in vivo*.

## Discussion

Breast cancer dramatically threatens the lives and health of women, and drug resistance makes the treatment of breast cancer challenging 4. Paclitaxel is now one of the commonly used first-line drugs in breast cancer chemotherapy 4. Exploring the specific mechanisms of paclitaxel resistance is helpful in providing new ideas for the clinical treatment of breast cancer. The chemotherapy of breast cancer is often accompanied by the onset of ferroptosis, and the increased resistance to ferroptosis often promotes chemoresistance in breast cancer cells. Nrf2, as a nuclear transcription factor, plays an important antioxidant role in cells, and its downstream antioxidant elements have an important ability of ferroptosis resistance 30, 31. Keap1 is an upstream regulator of intracellular Nrf2, and under stress, dissociation of Keap1 from Nrf2 promotes Nrf2 nuclear translocation, which in turn promotes the transcription of Nrf2 downstream factors 21. Therefore, focusing on the ability of the Keap1-Nrf2 signaling pathway that exerts intracellular resistance to ferroptosis could help to explore the specific mechanisms which breast cancer cells exhibit chemoresistance. In previous studies, miRNAs have been shown to be an important regulator of proliferation, migration, invasion, EMT phenotype and even chemoresistance in tumor cells 32-34. miR141-3p has been shown to regulate the Keap1-Nrf2 signaling pathway and promote tumor progression in previous studies 25, 35, 36. However, few studies have examined whether miR-141-3p has a role in promoting paclitaxel resistance in breast cancer. Given that, this present study provides an in-depth discussion of the important impact of miR-141-3p, an upstream regulator of Keap1-Nrf2 signaling pathway, on paclitaxel resistance in breast cancer cells.

We first analyzed the differentially expressed miRNAs in the GSE154255 dataset in the GEO database and extracted a potential miRNA (miR-141-3p) by taking the intersection of the 20 miRNAs in GSE154255 that are significantly highly expressed in breast cancer with the two miRNAs which can target Keap1. Meanwhile, by bioinformatics analysis we also observed that miR-141 is highly expressed and associated with poor prognosis in breast cancer. miR-141-3p was shown to have a role in targeting and regulating the Keap1-Nrf2 signaling pathway in previous studies 24, 35. Comparison of miR-141-3p expression in breast cancer cell lines MCF-7, MDA-MB-231 and normal breast cell line MCF-10A by using qRT-PCR showed that miR-141-3p was highly expressed in breast cancer cells. Dual luciferase reporter assay showed that miR-141-3p binds specifically to the 3'UTR of Keap1. The above evidence suggests that miR-141-3p may have an important regulatory role in the Keap-Nrf2 signaling pathway.

Subsequently, we overexpressed miR-141-3p in MCF-7 and MDA-MB-231 cells to observe the role of miR-141-3p in the regulation of Keap1-Nrf2 as well as its effect on paclitaxel resistance in breast cancer cells. Our results showed that overexpression of miR-141-3p inhibited the expression of Keap1 and promoted the expression of Nrf2, as well as significantly promoted paclitaxel resistance in breast cancer cells. These data suggest that modulation of the Keap1-Nrf2 signaling pathway by mir-141-3p may promote paclitaxel resistance in breast cancer cells.

To further explore the specific mechanism by which miR-141-3p promotes paclitaxel resistance in breast cancer cells, we utilized miR-141-3p inhibitors to inhibit mir-141-3p in breast cancer cells. As Keap1-Nrf2 plays an important role in cellular resistance to ferroptosis 37, 38, We focused on changes in SLC7A11-GSH-GPX4, an important ferroptosis resistance system, and ferroptosis levels after disrupting miR-141-3p in breast cancer cells. Our data suggest that targeted inhibition of miR-141-3p promoted Keap1 expression and suppressed Nrf2 expression and inhibited SLC7A11-GSH-GPX4, the downstream anti-ferroptosis pathway of Nrf2, in cancer cells. MDA is the final product of lipid peroxidation, and MDA content effectively reflects the level of ferroptosis in cells 39. JC-1 is a fluorescent probe widely used to detect mitochondrial membrane potential, and it is also an effective way to flank the response to ferroptosis by detecting the level of intracellular mitochondrial membrane potential 40. Our data suggest that targeted inhibition of miR-141-3p was able to further elevate MDA content and further decrease mitochondrial membrane potential at the treatment of paclitaxel in cancer cells. Meanwhile, inhibition of miR-141-3p significantly suppressed paclitaxel resistance and RSL3 tolerance in breast cancer cells. The above data suggested that inhibition of miR-141-3p may have suppressed paclitaxel resistance by promoting ferroptosis in breast cancer cells.

ML385 is a specific inhibitor of Nrf2. ML385 can specifically interacts with Nrf2 and significantly inhibits the transcriptional activity of Nrf2. In previous studies, ML385 exhibited good inhibition of Nrf2 and inhibited the progression of breast cancer cells 41, 42. Our results indicated that the inhibition of Nrf2 by ML385 significantly attenuated the promotion of ferroptosis resistance and paclitaxel resistance by miR-141-3p in breast cancer cells. This suggests that the action of miR-141-3p in facilitating ferroptosis resistance and paclitaxel resistance was induced by modulating Nrf2 in breast cancer cells.

To further validate the paclitaxel resistance role played by miR-141-3p in breast cancer, We constructed MDA-MB-231 cell lines with low-expression and overexpression of miR-141-3p by using lentivirus. Cancer cells were ectopically implanted subcutaneously in nude mice, and the tumor growth was observed in each group of mice under paclitaxel treatment. Overexpression of miR-141-3p significantly promoted paclitaxel resistance in breast cancer cells in mice, while inhibition of miR-141-3p significantly suppressed paclitaxel resistance in breast cancer cells in mice. This suggested that miR-141-3p has an important effect on paclitaxel resistance in breast cancer cells *in vivo*.

miR-141-3p has been found to have a role in promoting tumor progression in a variety of tumors, including promoting the progression of gastric, prostate, and ovarian cancers^24^, ^25^, ^43^. Meanwhile, miR-141-3p was also reported to have an important regulatory role on Keap1-Nrf2 signaling pathway. However, the effect of miR-141-3p on paclitaxel resistance in breast cancer has not yet been reported^24^. Here, we obtained miR-141-3p by screening miRNAs that are highly expressed in breast cancer with miRNAs that may bind to Keap1. And, miR-141-3p was experimentally observed to be able to attenuate ferroptosis by regulating the Keap1-Nrf2 signaling pathway, which in turn promoted paclitaxel resistance in breast cancer cells. Nevertheless, whether miR-141-3p has an effect on the ability of proliferation, migration, and invasion of breast cancer cells has not been explored in our work, and the effect of miR-141-3p on the resistance to other breast cancer chemotherapeutic drugs needs to be further discussed.

## Conclusion

In summary, miR-141-3p promotes the antioxidant capacity of Nrf2 in cells by targeting Keap1. Targeted inhibition of miR-141-3p significantly promoted ferroptosis by modulating the Keap1-Nrf2 signaling pathway in breast cancer cells under paclitaxel treatment, which in turn inhibited paclitaxel resistance in breast cancer cells. This study provides new ideas for the chemotherapy of breast cancer, but other roles played by miR-141-3p in breast cancer cells and its specific mechanisms are left to be further discussed.

## Figures and Tables

**Table 1:** The primers of qRT-PCR

Gene	sequence
U6	RT: GTCGTATCCAGTGCAGGGTCCGAGGTATTCGCAC
	Forward: CTCGCTTCGGCAGCACA
	Reverse: AACGCTTCACGAATTTGCGT
miR-141-3p	RT: GTCGTATCCAGTGCAGGGTCCGAGGTATTCGCACTGGATACGACCCATCT
	Forward: GAGCGCGTAACACTGTCTGGTA
	Reverse: ATCCAGTGCAGGGTCCGAGG
Keap1	Forward: GTGGCTGTCCTCAATCGTCTCC
	Reverse: CGCTTCGGATGGTGTTCATTGC
GAPDH	Forward: CCCCATACACAGTGTTAGCC
	Reverse: GAGTGATTTTCCCGTCC

**Table 2:** Antibody information of western blot

Antibody Name	Dilution	Cat Number
Rabbit anti-Keap1 Polyclonal antibody	1:2000	10503-2-AP, Proteintech
Rabbit anti-Nrf2 Polyclonal antibody	1:2000	16396-1-AP, Proteintech
Mouse anti-GPX4 Monoclonal antibody	1:1000	67763-1-Ig, Proteintech
Rabbit anti-SLC7A11 Polyclonal antibody	1:1000	36864-1-AP, Proteintech
Mouse anti-GAPDH Monoclonal antibody	1:3000	60004-1-Ig, Proteintech
anti-Alpha Tubulin Monoclonal antibody	1:3000	66031-1-Ig, Proteintech
Mouse anti-Histone H3 Monoclonal antibody	1:5000	68345-1-Ig, Proteintech
HRP-conjugated Affinipure Goat anti-Mouse IgG(H+L)	1:5000	SA00001-1, Proteintech
HRP-conjugated Affinipure Goat anti-Rabbit IgG(H+L)	1:5000	SA00001-2, Proteintech

## Abbreviations

Keap1: kelch-like ECH-associated protein 1
Nrf2: nuclear factor erythroid 2-related factor 2
SLC7A11: solute carrier family 7 member 11
GPX4: glutathione peroxidase 4
miRNA: microRNA
MDA: malondialdehyde
PTX: paclitaxel
GSH: glutathione
